# Percutaneous coronary intervention of anomalous right coronary artery arising from ascending aorta

**DOI:** 10.1093/ehjcr/ytz139

**Published:** 2019-08-13

**Authors:** Rajesh Vijayvergiya, Kewal Kanabar, Prashant Panda, Anupam Lal

**Affiliations:** 1 Department of Cardiology, Advanced Cardiac Centre, Postgraduate Institute of Medical Education and Research, Sector 12, Chandigarh, India; 2 Department of Radio-Diagnosis, Postgraduate Institute of Medical Education and Research, Sector 12, Chandigarh, India

## Case description

**Figure 1:**
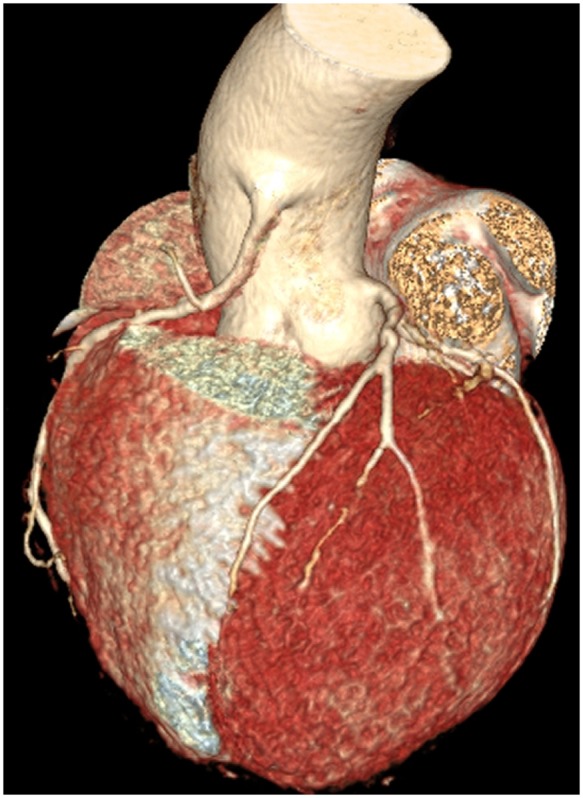
A volume-rendered computed tomography reconstructed image shows high take-off of the right coronary artery from the ascending aorta with a funnel-shaped ostium, while the left coronary artery is arising from the sino-tubular junction at the left aortic sinus.

**Figure 2:**
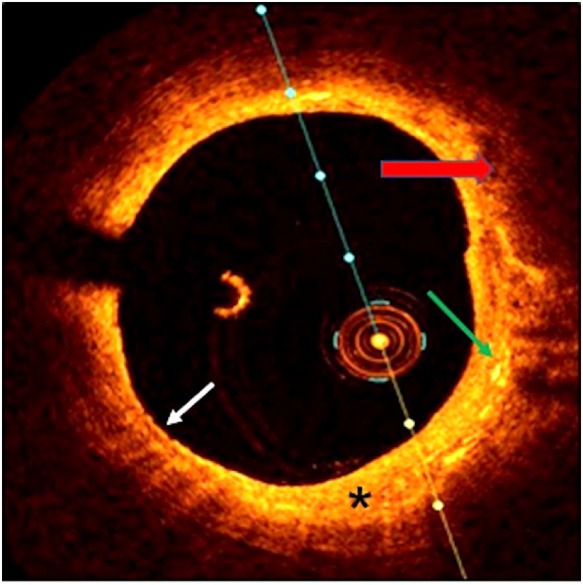
Optical coherence tomography image shows a thin-cap fibroatheroma (white arrow) with a dense lipid pool (red arrow), cholesterol crystals (green arrow), and fibrous tissue (asterisk) in the proximal right coronary artery.

A 50-year-old hypertensive male presented to the emergency room with unstable angina (rest angina Class IIB as per the Braunwald classification). Electrocardiogram showed pathological Q-waves and T-wave inversion in the inferior limb leads (Supplementary material online, *Figure S1*). Transthoracic echocardiogram showed no wall motion abnormality and a left ventricular ejection fraction of 60%. Coronary angiogram revealed normal left coronary artery, while the right coronary artery (RCA) could not be localized despite using various diagnostic catheters such as Amplatz Right (AR1), Amplatz Left (AL1), Judkins Right (JR), and an aortic root angiogram by a pigtail catheter (Supplementary material online, *Videos S1* and *S2*). A computed tomography coronary angiogram revealed an anomalous high origin of RCA from the ascending aorta, 3 cm above the sino-tubular junction on the right side (*Figure 1*) and significant stenosis of the proximal and distal RCA. The RCA was cannulated at the ascending aorta using a 6-Fr AL1 coronary guide catheter. There was a high take-off of RCA from the ascending aorta (Supplementary material online, *Video S3*) and diffuse disease in the proximal segment and from the distal segment to the posterior left ventricular (PLV) branch (Supplementary material online, *Video S4*). Optical coherence tomography (OCT) showed a fibro-fatty plaque with a thin-cap atheroma (*Figure 2* and Supplementary material online, *Video S5*). Two overlapping Xience Prime stents (Abbott Vascular, Santa Clara, CA, USA) of 3 × 38 mm and 2.75 × 38 mm were deployed in the distal RCA-PLV segment and a 4 × 33 mm Xience Prime stent was deployed in the proximal RCA with a good angiographic end-result (Supplementary material online, *Video S6*). Repeat OCT imaging after the intervention revealed well-apposed stent struts with no significant residual stenosis or edge dissection. He was discharged on Day 3 on dual antiplatelet therapy (aspirin 300 mg and clopidogrel 75 mg), along with atorvastatin 40 mg, metoprolol succinate 50 mg, and Telmisartan 80 mg. He remained asymptomatic during 18 months of clinical follow-up.

To the best of our knowledge, there are only five described cases with very high take-off of RCA from the ascending aorta above the sino-tubular junction on the right side.^1–4^ This is the first case where PCI with intravascular imaging was done for this rare anomaly.

## Supplementary material

Supplementary material is available at *European Heart Journal - Case Reports* online.

**Consent:** The author/s confirm that written consent for submission and publication of this case report including image(s) and associated text has been obtained from the patient in line with COPE guidance.

**Conflict of interest:** none declared.

## Supplementary Material

ytz139_Supplementary_Data
