# Supplementary figures and images for: Percutaneous coronary intervention of anomalous right coronary artery arising from ascending aorta
Source: Eur Heart J Case Rep. 2019 Aug 13;3(3):ytz139. doi: 10.1093/ehjcr/ytz139 (PMC6764536; doi:10.1093/ehjcr/ytz139)

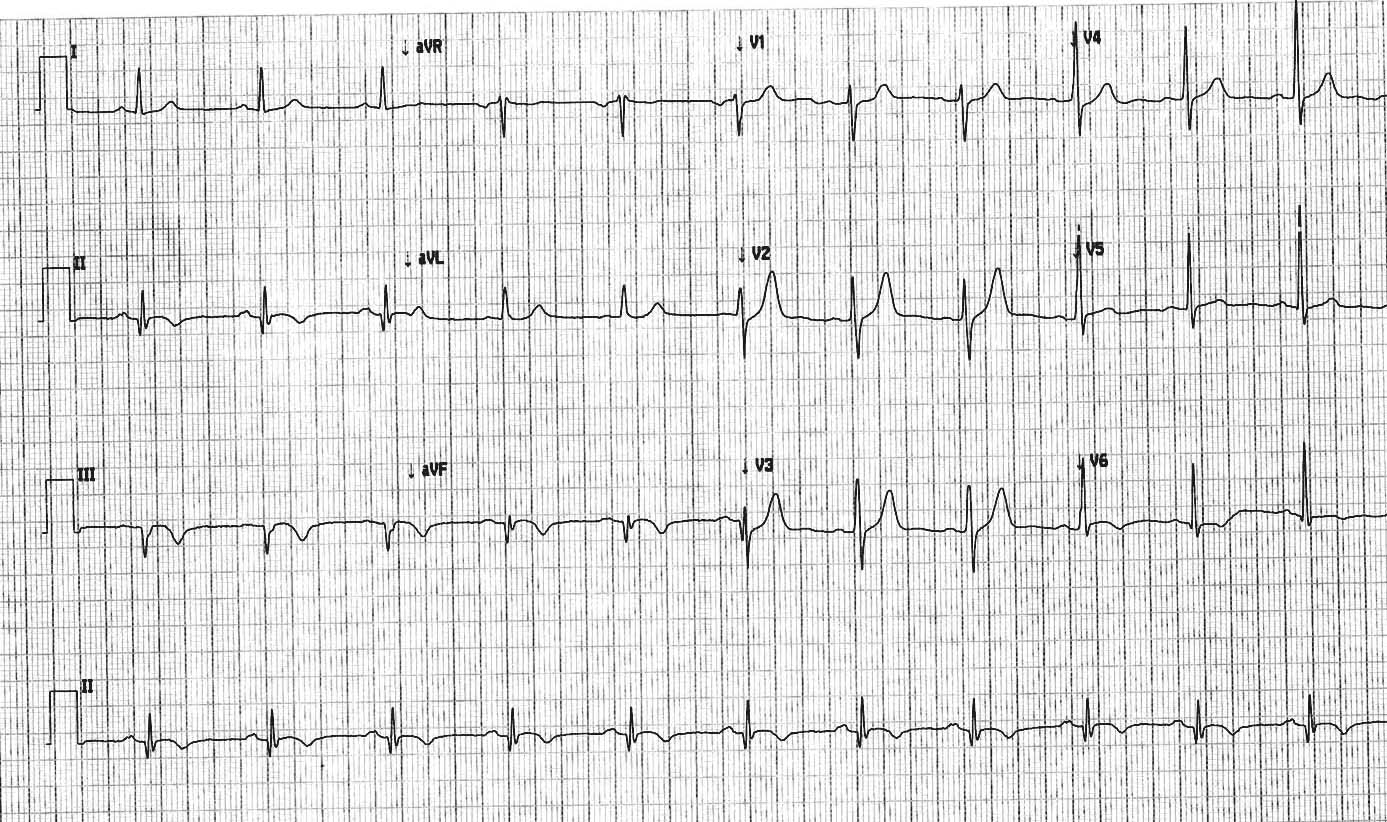

Supplement: ytz139_Supplementary_Data [file ytz139_supplementary_data.zip › ytz139-Suppl_data/Supplementary Material Figure S1.jpg]
